# Immunoregulatory Effects of Mitochondria Transferred by Extracellular Vesicles

**DOI:** 10.3389/fimmu.2020.628576

**Published:** 2021-02-09

**Authors:** Zhou She, Min Xie, Marady Hun, Amin Sheikh Abdirahman, Cuifang Li, Feifeng Wu, Senlin Luo, Wuqing Wan, Chuan Wen, Jidong Tian

**Affiliations:** Department of Pediatrics, The Second Xiangya Hospital, Central South University, Changsha, China

**Keywords:** mitochondria, extracellular vesicles, immune cell, immunoregulation, mesenchymal stem cells

## Abstract

Mitochondria participate in immune regulation through various mechanisms, such as changes in the mitochondrial dynamics, as metabolic mediators of the tricarboxylic acid cycle, by the production of reactive oxygen species, and mitochondrial DNA damage, among others. In recent years, studies have shown that extracellular vesicles are widely involved in intercellular communication and exert important effects on immune regulation. Recently, the immunoregulatory effects of mitochondria from extracellular vesicles have gained increasing attention. In this article, we review the mechanisms by which mitochondria participate in immune regulation and exert immunoregulatory effects upon delivery by extracellular vesicles. We also focus on the influence of the immunoregulatory effects of mitochondria from extracellular vesicles to further shed light on the underlying mechanisms.

## Introduction

As the energy storehouse of cells, mitochondria provide ATP for cellular activities and are, thus, indispensable organelles for cell survival. Numerous studies have shown that mitochondria not only serve as energy centers of cells, but also exert important immunoregulatory effects. They participate in immune function mainly through changes in the mitochondrial dynamics. They function as metabolic mediators of the tricarboxylic acid cycle and are involved in the production of reactive oxygen species (ROS) and in mitochondrial DNA damage, among others.

Extracellular vesicles are bilayer membranous substructures produced by various cell types. They contain cellular proteins, nucleic acids, and even organelles, which can reach all parts of the body through the circulation and other body fluids, thereby constituting an important tool for intercellular communication (1). By using extracellular vesicles, mitochondria can transfer between cells and act on specific target cells, thus achieving their biological functions. This article discusses the immunoregulatory effects of mitochondria, as well as the effects of mitochondria on target cells upon delivery by extracellular vesicles.

## Immunoregulatory Effects of Mitochondria

**Figure 1:**
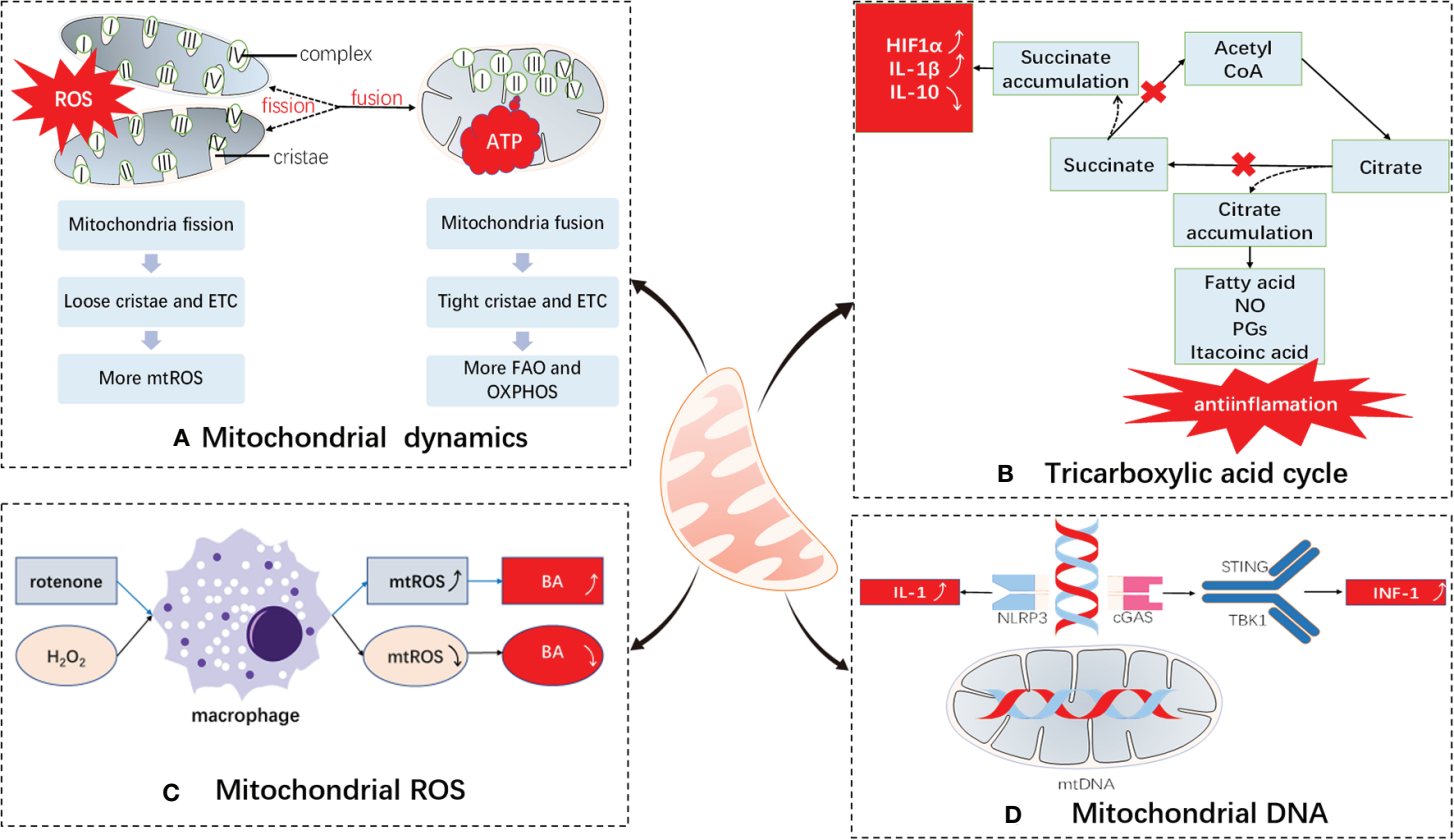
Immunomodulatory effects of mitochondria. Mitochondria undergo **(A)** dynamic changes: The electron transport chains of split and fused mitochondria are different and tend to produce ROS and ATP, respectively; **(B)** metabolic medium of the tricarboxylic acid cycle: blockade of the tricarboxylic acid cycle leads to accumulation of inflammatory substances; **(C)** ROS production: mitochondrial ROS are important bactericidal substances of macrophages; **(D)** DNA damage participates in immune regulation: mtDNAs participate in immune responses by serving as DAMPs. ETC, electron transport chain; BA, bactericidal; ROS, reactive oxygen species; FAO, fatty acid oxidation; OXPHOS, oxidative phosphorylation; NLRP3, NACHT, LRR, and PYD domain-containing protein 3; DAMPs, damage-associated molecular patterns; cGAS, cyclic GMP-AMP synthase; STING, stimulator of interferon genes; TBK1, TANK-binding kinase 1.

Mitochondria are cellular energy factories of eukaryotes and the metabolic centers of cells. They serve to produce ATP through the tricarboxylic acid cycle and electron transport chain and link a variety of metabolic pathways to maintain cellular energy homeostasis. However, an increasing number of studies have shown that mitochondria can also participate in immune regulation through a variety of mechanisms (2) (**Figure 1**).

### Changes in Mitochondrial Dynamics

Through continuous fission and fusion, mitochondria change their size, mass, and location within cells. These processes are summarized as mitochondrial dynamics (3). By regulating mitochondrial dynamics, cells can clear mitochondria that have lost their functions, repair damaged mitochondria, change the position of mitochondria, and ensure that sufficient energy supplies are provided, so as to respond to changes in the surrounding environment in a timely manner (4, 5).

In the case of immune cells, mitochondrial dynamics are mainly restricted to changes in the shape of the cristae to divert cellular functions. Fused mitochondria have larger cristae areas, with the cristae being closely linked, ensuring the patency of the electron transport chain, while at the same time mediating more efficient oxidative phosphorylation; on the contrary, cells with split mitochondria tend to utilize glycolysis for energy production (2). A previous study reports that naive T cells contain dissociated, circular mitochondria, and that their mitochondrial morphology changes through mitochondrial dynamics during activation, disconnecting the electron transport chain, increasing the production of ROS, and benefiting their own activation (6). Effector T cells tend to divide their mitochondria to form punctate structures, which leads to the dispersion of mitochondrial cristae, thus reducing the efficiency of the electron transport chain and promoting glycolysis. Mitochondrial fusion in memory T cells tends to form long column-shaped mitochondria, thereby enhancing the connection of the electron transport chain and improving the efficiency of oxidative phosphorylation and fatty acid oxidation. The mitochondrial intimal fusion protein, OPA1, is essential for the formation of memory T cells (7), which may help maintain their long-term survival. In addition, studies have shown that the fusion and fission of mitochondria may inhibit or promote the expression of interleukin (IL)-12 upon stimulation of toll-like receptors, which in turn induces T cells to secrete gamma interferon, thus promoting the polarization of T cells into Th1 cells (8, 9).

Mitochondrial dynamics can also affect the function of immune cells by changing the position of the mitochondria. In the process of T cell activation, mitochondria are dynamic and cooperate with kinesin to realize their movement to immunological synapses, to improve ATP supply and control the concentration of calcium ions to help T cells maintain an activated state (10, 11).

### Metabolic Mediators of the Tricarboxylic Acid Cycle

Through the tricarboxylic acid cycle and electron transport chain, mitochondria can complete oxidative phosphorylation, thus representing the energy utilization mode of most cells (12). In addition to energy production, some metabolites of the tricarboxylic acid cycle also serve as important mediators for regulating immune responses (13).

Previous studies have shown that there are two break points in the tricarboxylic acid cycle of M1 macrophages. One occurs in the step involving isocitrate dehydrogenase (IDH), which leads to an increase in citric acid, and the other occurs in an enzymatically catalyzed step involving succinate dehydrogenase, which leads to an increase in succinate production. On the one hand, citric acid is used to synthesize fatty acids, nitric oxide, prostaglandins, and itaconic acids. The former three are key substances required for the synthesis of anti-inflammatory factors by M1 macrophages. Itaconic acid can inhibit isocitrate lyase, which prevents the growth of bacteria, including Mycobacterium tuberculosis, and also plays an anti-inflammatory role (2, 6, 12, 14–16). On the other hand, succinic acid is an important pro-inflammatory factor. It can combine with hypoxia-inducible factor-1*α* to maintain its stability to promote the production of the inflammatory mediator, IL-1β. At the same time, it can also inhibit the production of the anti-inflammatory factor, IL-10 (12, 17–19). However, M2 macrophages are characterized by a fully functional tricarboxylic acid cycle, and do not produce too many inflammatory substances, which is in accordance with the anti-inflammatory effect of the M2 macrophages. In addition, researchers have reported that the tricarboxylic acid cycle can increase the production of interferons by increasing acetyl coenzyme A levels in the cytoplasm, which enhances the function of Th1 ancillary cells (20).

### Mitochondrial ROS Production

ROS are important defense molecules of innate immunity that are generated by mitochondria and NADPH oxidase. In the transfer process of a single electron, superoxide is formed as it collides with an oxygen molecule (O2) at a specific location (such as complexes I and III), resulting in ROS (21, 22). Initially, they have been proposed to kill bacteria directly by chemical action and were considered a by-product of monocyte respiration (23, 24). Further research has shown that mitochondrial ROS (mtROS) have important effects on immune regulation (22). Uncoupling protein 2 (UCP2) is a negative regulator of mtROS. UCP2-deficient mice exhibit stronger resistance to Toxoplasma gondii and *Salmonella typhimurium* infections, as well as macrophages with stronger cytokine-release potential (22, 25). Previously, researchers have also used rotenone to inhibit the electron transport chain of macrophages, resulting in a significant increase in mtROS. It has been reported that the production of IL-1β by macrophages is significantly increased in a dose-dependent manner (26). On the contrary, when activated macrophages are exposed to hydrogen peroxide, their anti-bacterial activity decreases significantly (27).

For adaptive immune cells, mtROS are also important mediators of associated immune functions. It was found that mtROS are essential for activating T cell nuclear factors and T cells to produce IL-2 (28). Moreover, after inhibiting glycerol-3-phosphate dehydrogenase 2 (GPD2, an enzyme that produces mtROS) in T cells, IL-2 production is reduced (2, 29). Early B cells undergo somatic hypermutation and class-switch recombination to change the properties of the antigen receptor and immune globulin molecules, and eventually differentiate into memory B cells or plasma cells. Previous studies have shown that increasing mitochondrial mass and mtROS of B cells can inhibit heme synthesis, relieve the antagonistic effect of BACH2 (antigen category conversion for necessary transcription factors), and enhance class-switch recombination (2, 6).

### Mitochondrial DNA Damage

Mitochondrial DNA is mainly involved in immunity as a damage-related model molecule, more specifically, a damage-associated molecular pattern (DAMP) (30). During apoptosis, mitochondria release oxidized mitochondrial DNA and bind to NLRP3 inflammatory bodies, thus promoting the activation of downstream cell apoptotic protease-1 and the production of IL-1 (2, 31). Thus, mitochondrial DNA establishes a link between mitochondrial damage and apoptotic protease-1 (32).

Another substance binding to the mitochondrial DNA is cyclic guanosine monophosphate-adenosine monophosphate (cGAMP) synthase (cGAS) (33, 34). When mitochondrial DNA enters the cytoplasm, it combines with cGAS to promote the formation of cGAMP, which serves as a second messenger, and acts in conjunction with downstream mediators, such as stimulator of interferon genes (STING) and TANK binding kinase-1 (TBK1), and promotes the formation of type 1 interferons, thus playing an important anti-viral role (35–37).

## Immunomodulatory Effects of Extracellular Vesicles

Extracellular vesicles are a type of bilayer membrane vesicles released by cells, with diameters ranging from 40 to 2000 nm. They contain a variety of substances, including DNA, proteins, lipids, and organelles, among others, which can be exchanged between cells with the help of the internal environment (1, 38–41). Material exchange through extracellular vesicles provides the following advantages: 1. Protection: The membrane structure of extracellular vesicles can protect the contents from degradation; 2. Targeting: The unique markers on the surface of extracellular vesicles can bind to the corresponding receptors on the target cells to achieve specific targeting; 3. Enrichment: The contents of the extracellular vesicles are released around or inside the target cells, which can locally increase the concentration of the substances released (42). Owing to their unique contents and the ability to act on intercellular communication, extracellular vesicles can be used as disease markers, therapeutic targets, and drug carriers, and have great potential in the diagnosis, monitoring, and treatment of diseases (1, 39, 41, 43–46). For example, in cardiovascular diseases, extracellular vesicles may promote the development and progression of atherosclerosis by promoting initial lesion formation, intravascular calcification, unstable plaque progression and post-rupture thrombosis, while extracellular vesicles released from stem cells have the function of promoting capillary angiogenesis, reducing infarct size, and oxidative stress (47); in metabolic diseases, extracellular vesicles may regulate insulin resistance, or macrophage phenotype, thus affecting disease progression (48, 49).

Extracellular vesicles play an important role in immunity by transferring protein components, such as cytokines, major histocompatibility complexes (MHC), and annexin (50). Dendritic cells, macrophages, and mast cells release extracellular vesicles containing MHC-II molecules, which serve to transfer them to target cells to influence antigen presentation and activate cellular immunity (50–52). It has been shown that macrophages infected with Mycobacterium tuberculosis can release MHC-II and Mycobacterium tuberculosis antigen, thus activating T cells and cellular immunity (53, 54).

In addition to proteins, extracellular vesicles can also carry miRNAs with various immunomodulatory effects (1). In a mouse model of myocardial ischemia and reperfusion, mesenchymal stromal cells secreted exosomes loaded with miRNA-182, which were shown to target the TLR4/NF-*κ*B/PI3K/Akt pathway, stimulate macrophages to differentiate into the M2 type, play an anti-inflammatory role, and reduce damage (55). In addition to macrophages, exosomes from mesenchymal stem cells have been reported to carry miR-let7, which can induce M2 macrophage transformation through the HMGA2/NF-*κ*B pathway and ameliorate atherosclerosis (56). In a mouse model of septicemia, miR-146a is shown to target the TRAF6 pathway, consequently promoting M2 macrophage polarization and prolonging the survival time of mice (57).

## Immunoregulatory Effects of Mitochondria Transferred by Extracellular Vesicles

**Figure 2:**
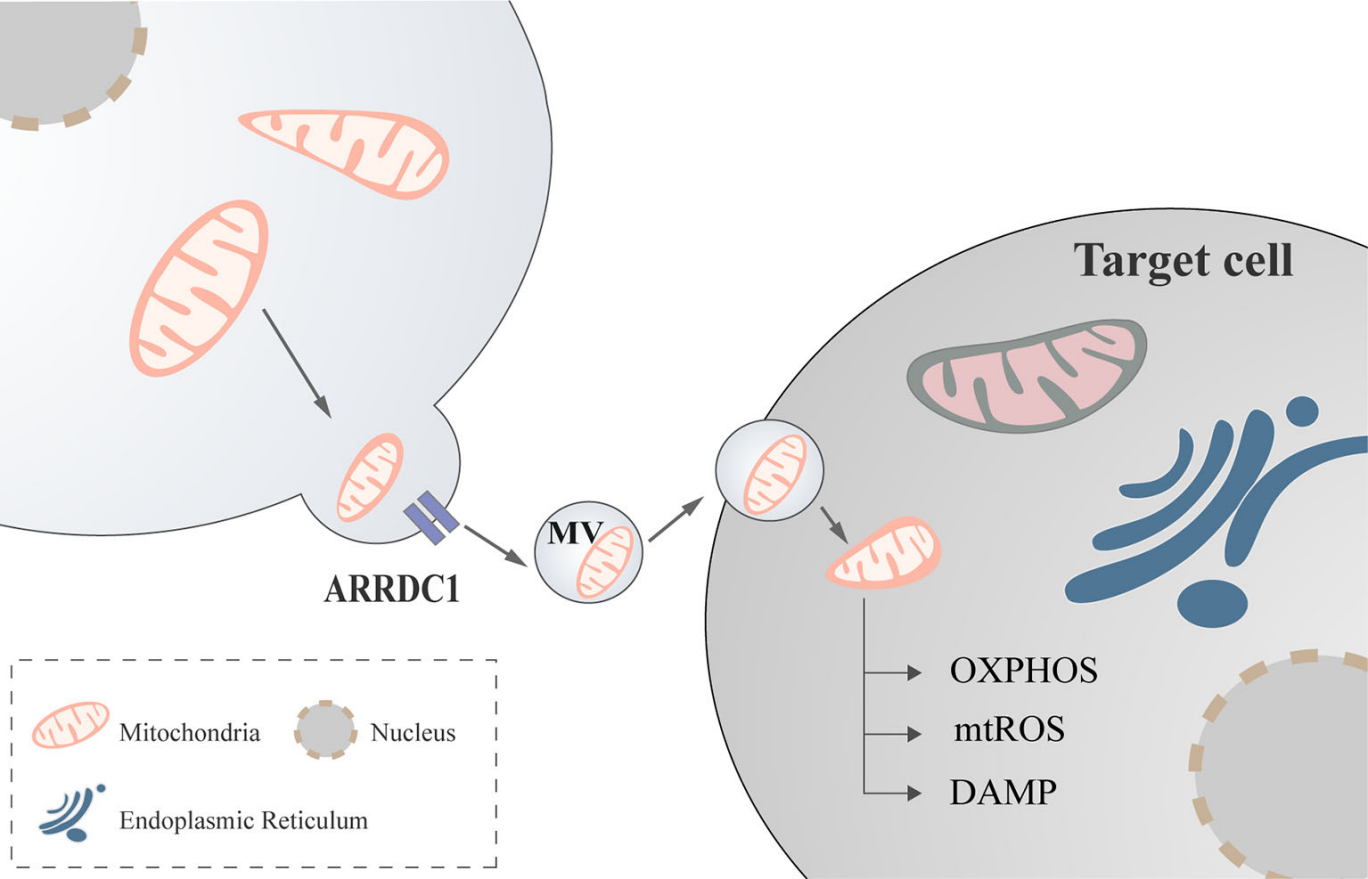
Mitochondria regulate immune cell *via* extracellular vesicles. Mediated by ARRDC1, mitochondria communicate between cells with the help of extracellular vesicles to modulate cellular functions. ARRDC1, arrestin domain-containing protein 1.

Mediated by arrestin domain-containing protein 1(ARRDC1), cells can release extracellular vesicles containing mitochondria (58, 59), a process that may be mediated by CD-38 (60), but the exact form remains to be investigated (**Figure 2**).

Previous studies identified mitochondria to be enclosed in extracellular vesicles, based on which they were considered to be related to the pathogenesis of certain diseases and disease markers (**Table 1**). For example, some researchers extracted extracellular vesicles containing mitochondria from blood samples of patients with systemic lupus erythematosus, and the concentration of those vesicles was shown to be related to disease activity and the concentration of the inflammatory factors. Further research revealed that extracellular vesicles carrying mitochondria may be involved in the pathogenesis of lupus erythematosus (62). Similarly, some researchers extracted extracellular vesicles from the plasma of patients with melanoma, which contained mitochondrial proteins. The latter may become unique marker proteins of extracellular vesicles associated with melanoma, as they are different from canonical extracellular vesicles (61).

**Table 1:** Mitochondrial immunomodulatory effects upon delivery by extracellular vesicles.

Origin of EVs	Target cells	Direct effects	Indirect effects	Reference
Patient melanoma metastatic tissues	–	Biomarker of tumor cells	–	(61)
Blood samples of patients with SLE	–	Promote inflammation	Related to disease activity	(62)
BM-MSCs	Alveolar epithelium	Increase in ATP levels	Protect against ALI	(63)
hBM-MSCs	Macrophage	Increase in ATP and ROS levels	React to Oxidative stress	(64)
MSCs	Macrophage	Enhance bioenergetics	Regulate macrophage functions	(58)
Astrocytes	–	Increase in ATP levels	–	(65)
Astrocytes	Neurons	Increase in ATP levels	Promote survival and plasticity	(60)
MDRCs	T cells	Co-localize with the mitochondrial network of T cells	Increase ROS generation	(66)
LPS-stimulated monocytic cells	Endothelial cells	Mediators of communication	Promote inflammation	(67)
Platelets	Neutrophils	Substrate for the bactericidal secreted phospholipase A2-IIA	Regulate immune reactions	(68)

BM-MSCs, Bone marrow-derived stromal cells; hBM-MSCs, human bone marrow-derived stromal cells; MSCs, mesenchymal stem cells; MDRCs, airway myeloid-derived regulatory cells, ALI, acute lung injury.

As mentioned above, extracellular vesicles themselves constitute an important tool for intercellular communication, along with immunomodulatory effects (1, 40, 69). In addition, mitochondria can participate in immune regulation in various ways and exert direct or indirect regulatory effects on immune cells. For example, they affect the selective differentiation of B cells and the production of antibodies (70). When T cells are activated, their mitochondria assemble into specialized structures to produce sufficient amounts of one-carbon units (71). Therefore, it is worth exploring whether mitochondria play a role in immune regulation through extracellular vesicles.

Transferred by extracellular vesicles, mitochondria can regulate the function of other cells, such as alveolar epithelial cells, macrophages, nerve cells (neurons), neutrophils, and endothelial cells, and complete the communication between cells. Most transferred mitochondria achieve their functions in terms of regulation, protection, and recovery by increasing oxidative phosphorylation/energy production in target cells. For example, bone marrow-derived mesenchymal stem cells can transfer mitochondria to alveolar epithelial cells through extracellular vesicles, consequently improving energy production by epithelial and surrounding cells, restoring the function of type II alveolar epithelial cells to produce alveolar active substances, thus protecting alveolar epithelial cells in the case of acute lung injury, and improving the survival rate of mice with acute lung injury stimulated by lipopolysaccharide (63). However, how do transferred mitochondria protect the lungs by regulating immunity? Using a model of acute respiratory distress, researchers have found that mesenchymal stem cells could use extracellular vesicles to transfer mitochondria to alveolar macrophages. This led to an improvement in the oxidative phosphorylation ability of alveolar macrophages, and to their differentiation into the anti-inflammatory M2 type. It further served to enhance the phagocytic capacity of M2-type alveolar macrophages and reduce the secretion of TNF-α, thus reducing lung inflammation (64). In addition, studies have found that extracellular vesicles derived from mesenchymal stem cells could transfer mitochondria to macrophages and fuse with the mitochondrial network of macrophages, thus increasing ATP production (58).

The transported mitochondria are required to be functional (60, 63, 64). After a stroke, astrocytes use extracellular vesicles to transfer mitochondria to neurons through a CD38-dependent mechanism, which can protect cells from hypoxia and glucose deprivation, and improve the plasticity of cells after injury. It is worth exploring whether CD38 is also expressed in immune cells. In this series of experiments, no evidence was found that inhibition of CD38 could affect the release of mitochondria from immune cells; therefore, further research and discussion are warranted (60).

Moreover, platelets have been described to secrete vesicular mitochondria, which are decomposed by phospholipase A2-IIA (sPLA2-IIA) after being absorbed by neutrophils, followed by the release of mitochondrial DNA, lysophospholipids, and fatty acids. These substances can be used as damage-related model molecules to stimulate neutrophil activation and promote their pro-inflammatory responses (68). By serving as an energy source, the amount of energy provided by mitochondria is also a means of regulating an immune response. As mentioned above, in a disease model of acute respiratory distress syndrome, after the mitochondria derived from mesenchymal stem cells had entered the alveolar macrophages, they could enhance the oxidative phosphorylation capacity and promote the anti-inflammatory expression profile and phagocytic functions of macrophages, and reduce lung inflammation (64). Moreover, the mitochondrial network that is integrated into T cells was shown to affect the mitotic processes of T cells, highlighting another means by which extracellular vesicle mitochondria exert their effects (66).

In order to study the effect of activated monocyte vesicles on endothelial cell inflammation, Puhm et al. stimulated monocytes with lipopolysaccharide to produce extracellular vesicles containing mitochondria. These vesicles could induce type I interferon and tumor necrosis factor responses in endothelial cells. However, clearing these mitochondria or directly inhibiting the function of mitochondria in monocytes (pyruvate inhibition or use of a reactive oxygen scavenger to reduce ROS) could reduce the inflammatory effect of these vesicles on endothelial cells (67). This experiment highlighted that mitochondria in extracellular vesicles can stimulate the production of inflammatory responses and influence immune regulation.

## Future Perspectives

In conclusion, mitochondria not only supply energy to the cells, but also participate in immune regulation, directly or indirectly, through a variety of mechanisms. They are important immune-regulatory centers that have been widely studied. Moreover, mitochondrial dysfunction is also associated with many autoimmune diseases (72–74). The immune regulation of extracellular vesicles is a novel and important research hotspot. With the help of extracellular vesicles, mitochondria allow for communication between cells and better participation in the regulation of the immune system. These findings provide a direction for future extracellular vesicle-based or mitochondrial precision therapies. Although the ability of mitochondria to regulate immune cells through extracellular vesicles has been highlighted, there are still many unknown mechanisms that need to be addressed in future studies.

## Author Contributions

ZS and MX prepared the table and figure. ZS, MH, AA, CL, FW, SL, WW, JT, and CW drafted the manuscript. ZS and CW edited and revised the manuscript. All authors contributed to the article and approved the submitted version.

## Funding

This work was supported by grants from the Natural Science Foundation of Hunan Province in China (grant no. 2019JJ40413), the National Natural Science Foundation of China (grant no. 82070758), and Hunan Provincial Key R&D Program Project (grant no. 2020SK2084).

## Conflict of Interest

The authors declare that the research was conducted in the absence of any commercial or financial relationships that could be construed as a potential conflict of interest.
